# Supplementary material for: SARS‐CoV‐2 outbreak investigation in a German meat processing plant
Source: EMBO Mol Med. 2020 Oct 27;12(12):e13296. doi: 10.15252/emmm.202013296 (PMC7646008; doi:10.15252/emmm.202013296)
Supplement: Supplementary file 1 — Appendix [file EMMM-12-e13296-s001.pdf]

Appendix

SARS-CoV-2 outbreak investigation in a German meat processing plant

Thomas Günther[1§], Manja Czech-Sioli[2], Daniela Indenbirken[1], Alexis Robitaille[1], Peter Tenhaken[3], Martin Exner[4], Matthias Ottinger[5], Nicole Fischer[2*], Adam Grundhoff[1§*], Melanie M. Brinkmann[6,7*]

This PDF file includes:

Appendix Figures S1 to S3
Appendix Tables S1to S6

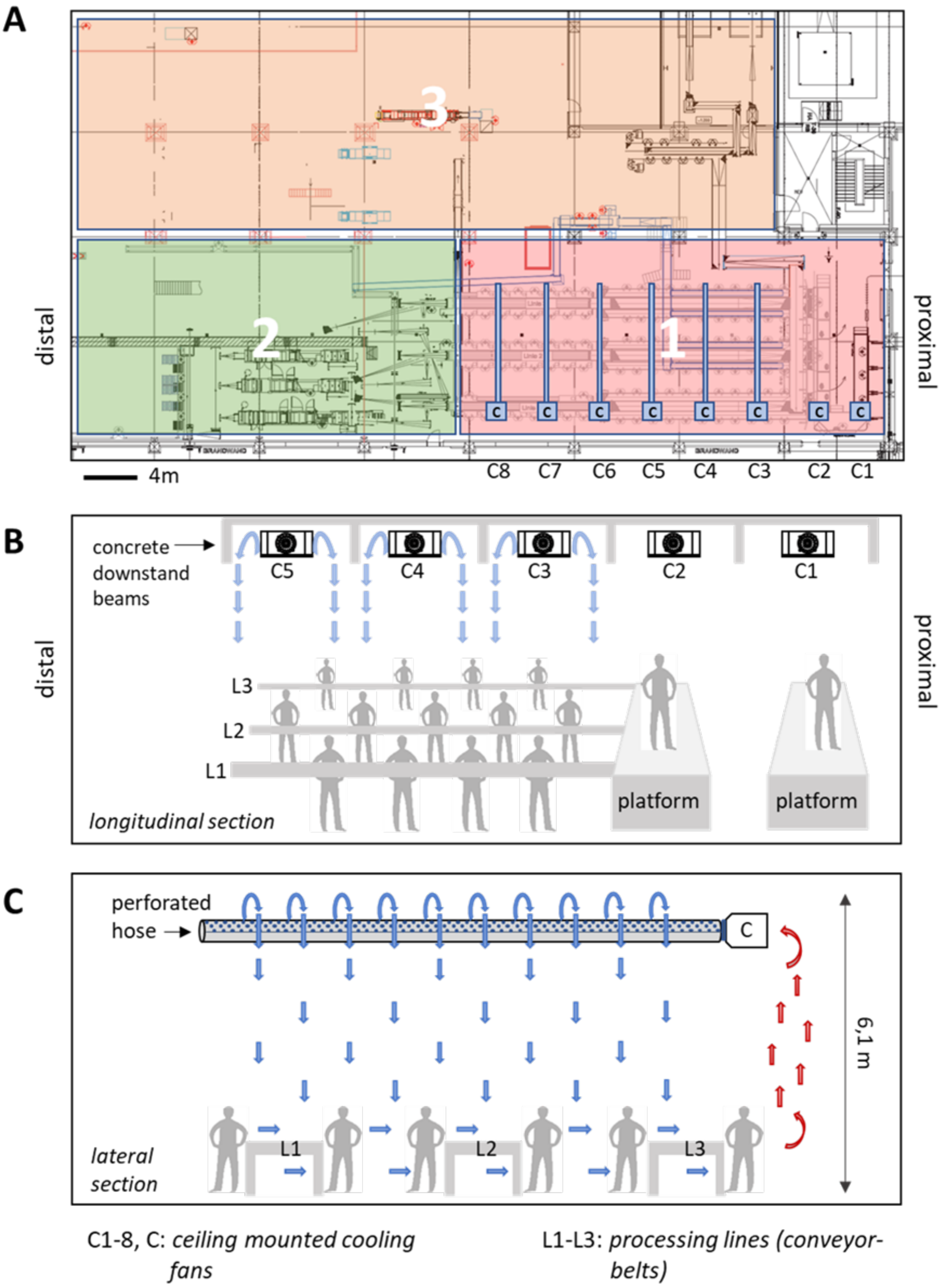

Appendix Fig. S1. Schematic layout and air-flow conditions of beef processing plant in MPP-R.

(A) Schematic layout of the beef processing plant. Beef halves enter the processing plant on the proximal side (area 1, red). In the section cooled by ceiling mounted cooling fans 1 (C1) and 2 (C2), beef halves are cut in quarters. Quarters are then translocated onto three processing lines (conveyor-belts, L1-3). L1-3 are moving in proximal-distal direction. Beef quarters are further processed in the section of cooling fans C3-5 (deboning), and finer cuts (shearing) are performed in section of cooling fans C6-8. Towards the distal part of the plant (area 2, green), the beef is packed into consumer packaging and packaging is sealed. Area 3 (orange) is used for weighing and packaging consumer units into boxes. Boxes are placed on pallets for shipping. Index case B1 worked in area 1 (red). Air conditioning units in area 1 are cooling recirculated air without filters (C1-8). C3-8 are connected to a perforated hose directed towards area 3 whereas C1 and C2 lack a hose. The air exchange rate value for the entire beef processing plant is <1 (i.e., it takes more than one hour to replace the air). (B) Schematic longitudinal section in distal-proximal direction of area 1. Beef halves are cut on platforms in the section cooled by cooling fans C1 and C2. C1 and C2 are operating without hose and turn on only when temperatures rise above 10°C. Cooled air is expelled through the hall up to a lateral distance of approximately 12 meters. Cooling fans 3-8 are operating permanently and are expelling cooled air through attached perforated hoses. (C) Schematic cross-section in the axis of one cooling fan/perforated hose. Cooling fan is expelling cooled, recirculated, unfiltered air into attached hoses. Hoses are perforated in the upper half thereby expelling air towards the ceiling. Concrete down stand beams are guiding the cool air downwards. The resulting air flow is resembling a laminar flow of cooled air from ceiling to the working area at the level of the conveyor-belt processing lines.

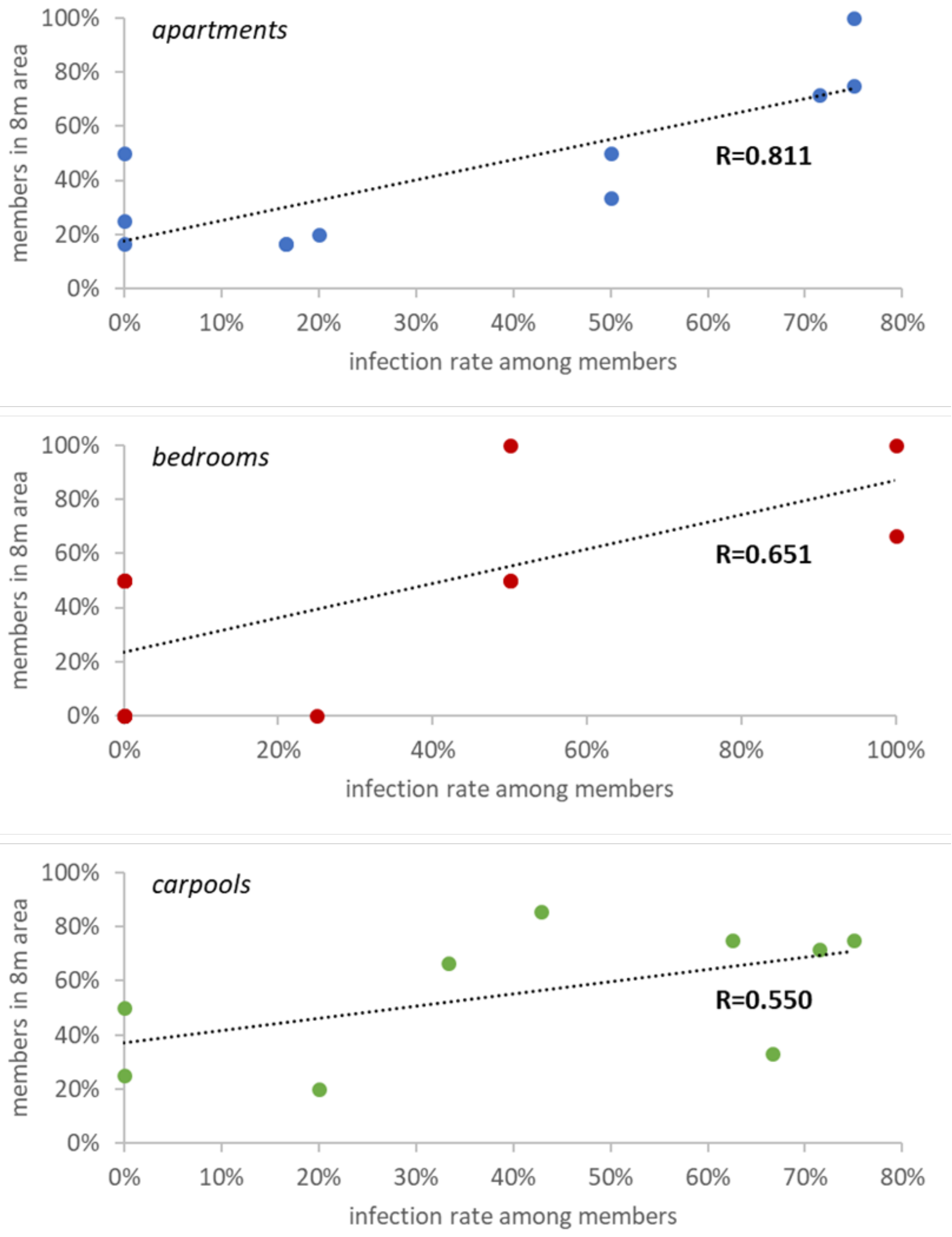

Appendix Fig. S2. Positive correlation between infection rates and work location in shared apartments, bedrooms and carpools.
For each apartment (top panel), bedroom (center panel) or carpool (bottom panel), plots show the percentage of positive unit members (x-axis) and the percentage unit members with fixed work stations in an 8 m area around the index case (y-axis). Individual values are given in Appendix Table SIII. Pearson correlation coefficients (R) are shown next to linear regression curves (dotted lines).

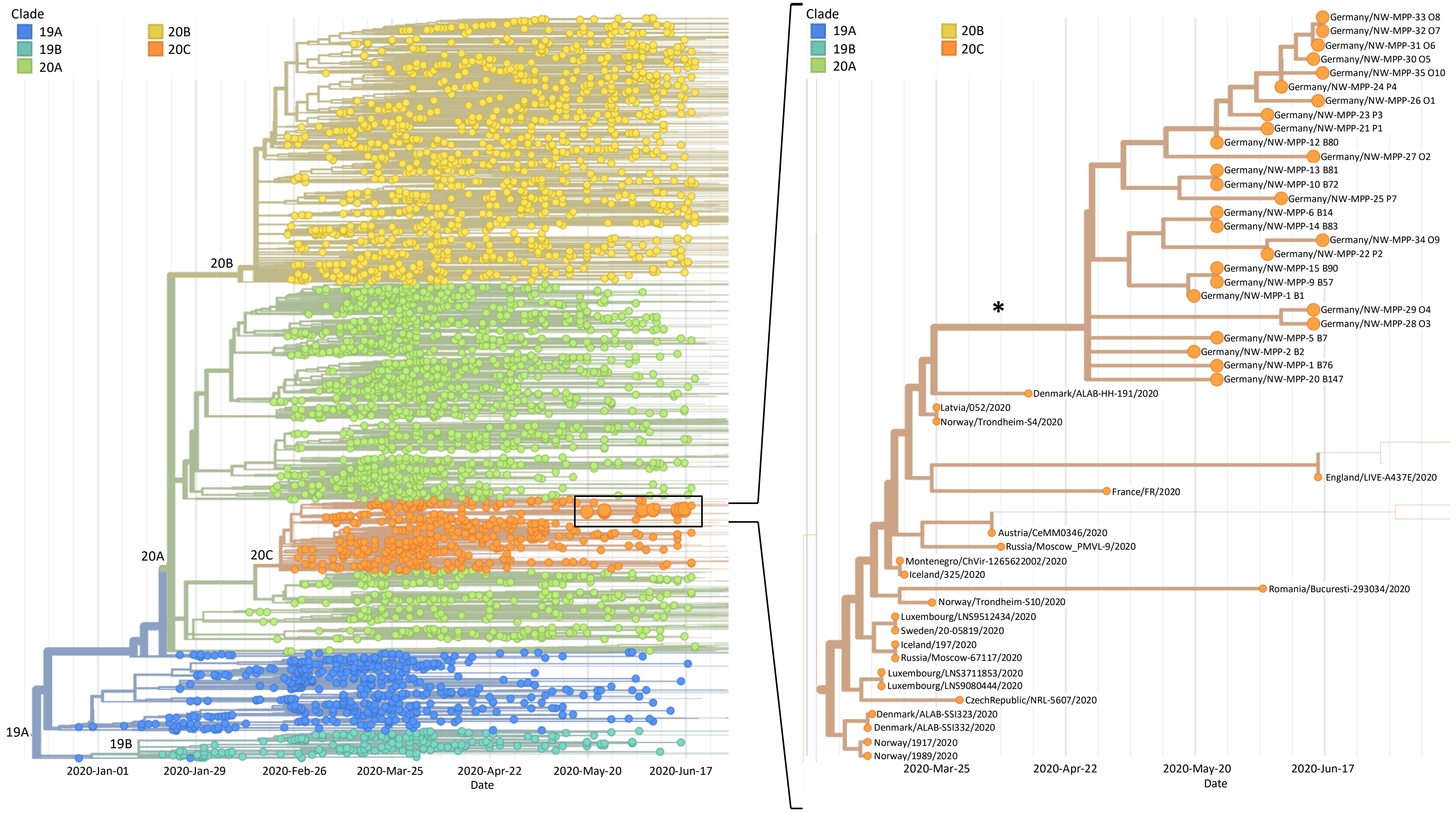

Appendix Fig. S3. Time-based phylogenetic analysis of MPP-R samples within the context of European SARS-CoV-2 isolates. Samples were analyzed and visualized within the phylogenetic context by nextstrain (nextstrain.org) using data available through GISAID (gisaid.org). Shown are 4051 of 5108 genomes sampled in Europe between Feb 2020 and Jun 2020 (left panel). MPP-R samples are indicated as enlarged circles in the rectangle. The branch containing MPP-R samples as well as related samples are depicted with GISAID identifier as well as the respective MPP-R sample ID in the right panel. The branch introducing the signature mutations C6406T and G18792A is marked by an asterisk.

Appendix Table S1. Positive test result dates and viral genotypes among MPP-R workers.

			nucleotide variant	C241T	C1059T	A1925C	C3037T	C5672T	C5796T	C6406T	A7394G	C7735T	C8139T	T10516C	G13812T	C14408T	C16049T	G17266A	G17266T	C18744T	G18972A	G21423T	A23403G	G24095T	G25563T	C28728T	G29543T	T29582C
			genomic region	n.c.	ORF1a; ORF1ab	ORF1a; ORF1ab	ORF1a; ORF1ab	ORF1a; ORF1ab	ORF1a; ORF1ab	ORF1a; ORF1ab	ORF1a; ORF1ab	ORF1a; ORF1ab	ORF1a; ORF1ab	ORF1a; ORF1ab	ORF1ab	ORF1ab	ORF1ab	ORF1ab	ORF1ab	ORF1ab	ORF1ab	ORF1ab	S	S	ORF3a	ORF9	n.c.	ORF10
			aa exchange	n.a.	T265I	T554P	silent	P1803S	T1844I	V2047V	I2377V	I2490I	S2625F	silent	M4516I	P4715L	T5262I	D5668N	D5668Y	silent	silent	R7053S	D614G	A845S	Q57H	A152V	n.a.	F9L
sample id	positive test date	GISAID id[a]	consensus genotype[b]	variant frequencies[c]																								
				C241T	C1059T	A1925C	C3037T	C5672T	C5796T	C6406T	A7394G	C7735T	C8139T	T10516C	G13812T	C14408T	C16049T	G17266A	G17266T	C18744T	G18972A	G21423T	A23403G	G24095T	G25563T	C28728T	G29543T	T29582C
B1	2020-05-20	hCoV-19/Germany/NRW-MPP-1/2020	protoype	100%	100%	0%	100%	0%	0%	100%	0%	0%	0%	0%	0%	99%	0%	0%	0%	0%	99%	0%	100%	0%	100%	0%	0%	0%
B2	2020-05-20	hCoV-19/Germany/NRW-MPP-2/2020	variant type 1	100%	100%	0%	100%	0%	0%	100%	0%	100%	0%	0%	0%	100%	0%	0%	0%	0%	100%	0%	100%	0%	100%	0%	0%	0%
B5	2020-05-25	hCoV-19/Germany/NRW-MPP-3/2020	protoype	100%	100%	0%	100%	0%	0%	100%	0%	0%	0%	0%	0%	99%	0%	0%	0%	0%	100%	0%	100%	42%	100%	0%	0%	0%
B6	2020-05-25	hCoV-19/Germany/NRW-MPP-4/2020	protoype	100%	100%	0%	100%	0%	0%	100%	0%	0%	0%	0%	0%	99%	0%	0%	0%	0%	100%	0%	100%	0%	100%	0%	0%	0%
B7	2020-05-25	hCoV-19/Germany/NRW-MPP-5/2020	variant type 2	100%	100%	0%	100%	100%	0%	100%	0%	0%	0%	97%	0%	100%	0%	0%	0%	0%	100%	0%	100%	0%	100%	0%	0%	0%
B14	2020-05-25	hCoV-19/Germany/NRW-MPP-6/2020	protoype	100%	100%	0%	100%	0%	0%	100%	0%	0%	0%	0%	0%	100%	0%	0%	0%	0%	100%	0%	100%	0%	100%	0%	0%	0%
B24	2020-05-25	hCoV-19/Germany/NRW-MPP-7/2020	variant type 3	100%	100%	0%	100%	0%	0%	100%	100%	0%	0%	0%	0%	100%	0%	0%	0%	1%	100%	0%	100%	0%	100%	0%	0%	0%
B28	2020-05-27																											
B33	2020-05-27																											
B54	2020-05-25																											
B55	2020-05-25	hCoV-19/Germany/NRW-MPP-8/2020	protoype	100%	100%	0%	99%	0%	0%	100%	0%	0%	0%	0%	0%	100%	0%	0%	0%	0%	100%	0%	100%	0%	100%	0%	0%	0%
B57	2020-05-25	hCoV-19/Germany/NRW-MPP-9/2020	protoype	100%	100%	0%	100%	0%	0%	100%	0%	0%	0%	0%	0%	100%	0%	0%	0%	0%	100%	0%	100%	0%	100%	0%	0%	0%
B66	2020-06-03																											
B67	2020-05-27																											
B68	2020-05-25																											
B72	2020-05-25	hCoV-19/Germany/NRW-MPP-10/2020	protoype	100%	100%	0%	100%	0%	0%	100%	0%	0%	0%	0%	0%	100%	0%	0%	0%	0%	100%	0%	100%	0%	100%	0%	0%	0%
B76	2020-05-25	hCoV-19/Germany/NRW-MPP-11/2020	variant type 4	100%	100%	0%	99%	0%	0%	100%	0%	0%	0%	0%	0%	100%	0%	0%	0%	0%	100%	0%	100%	0%	100%	100%	0%	0%
B80	2020-05-25	hCoV-19/Germany/NRW-MPP-12/2020	protoype	100%	100%	0%	99%	0%	0%	100%	0%	0%	0%	0%	0%	100%	0%	0%	0%	0%	100%	0%	100%	0%	100%	0%	0%	0%
B81	2020-05-25	hCoV-19/Germany/NRW-MPP-13/2020	protoype	100%	100%	0%	100%	0%	0%	100%	0%	0%	0%	0%	0%	100%	0%	0%	0%	0%	100%	0%	100%	0%	100%	0%	0%	0%
B83	2020-05-25	hCoV-19/Germany/NRW-MPP-14/2020	protoype	100%	100%	0%	100%	0%	0%	100%	0%	0%	0%	0%	0%	100%	0%	0%	0%	0%	100%	0%	100%	0%	100%	0%	0%	0%
B86	2020-06-03																											
B90	2020-05-25	hCoV-19/Germany/NRW-MPP-15/2020	protoype	100%	100%	0%	100%	0%	0%	100%	0%	0%	0%	4%	0%	100%	0%	0%	0%	0%	99%	0%	100%	0%	100%	0%	0%	0%
B93	2020-05-25	hCoV-19/Germany/NRW-MPP-16/2020	protoype	100%	100%	0%	100%	0%	0%	100%	0%	0%	0%	0%	0%	100%	0%	0%	0%	0%	100%	0%	100%	0%	100%	0%	0%	0%
B98	2020-05-25	hCoV-19/Germany/NRW-MPP-17/2020	protoype	100%	100%	0%	100%	0%	0%	100%	0%	0%	0%	0%	0%	100%	0%	0%	0%	0%	100%	0%	100%	0%	100%	0%	0%	0%
B105	2020-05-27																											
B108	2020-06-03																											
B113	2020-05-25	hCoV-19/Germany/NRW-MPP-18/2020	protoype	100%	100%	0%	100%	0%	0%	100%	0%	0%	0%	0%	0%	100%	0%	0%	0%	0%	100%	0%	100%	0%	100%	0%	0%	0%
B120	2020-05-25	hCoV-19/Germany/NRW-MPP-19/2020	protoype	100%	100%	0%	100%	5%	0%	100%	0%	0%	0%	0%	0%	100%	0%	0%	0%	0%	100%	0%	100%	0%	100%	0%	0%	0%
B138	2020-06-03																											
B143	2020-05-25																											
B147	2020-05-25	hCoV-19/Germany/NRW-MPP-20/2020	variant type 5	100%	100%	0%	100%	0%	0%	100%	0%	0%	0%	0%	0%	100%	0%	0%	0%	100%	100%	0%	100%	0%	100%	0%	0%	0%
O1	2020-06-16	hCoV-19/Germany/NRW-MPP-26/2020	protoype	100%	100%	0%	100%	0%	0%	n.a.	0%	0%	0%	0%	0%	100%	0%	0%	0%	0%	99%	0%	100%	0%	100%	0%	0%	0%
O2	2020-06-15	hCoV-19/Germany/NRW-MPP-27/2020	variant type 8	100%	100%	0%	100%	0%	0%	100%	0%	0%	0%	0%	0%	100%	0%	0%	100%	0%	100%	0%	100%	0%	100%	0%	0%	0%
O3	2020-06-15	hCoV-19/Germany/NRW-MPP-28/2020	variant type 9	100%	100%	0%	100%	0%	0%	100%	0%	0%	0%	0%	20%	100%	0%	0%	0%	0%	100%	53%	100%	0%	100%	0%	53%	53%
O4	2020-06-15	hCoV-19/Germany/NRW-MPP-29/2020	variant type 10	100%	100%	0%	100%	0%	0%	100%	0%	0%	0%	0%	20%	100%	0%	0%	0%	0%	100%	48%	100%	0%	100%	0%	54%	53%
O5	2020-06-15	hCoV-19/Germany/NRW-MPP-30/2020	protoype	100%	100%	0%	100%	0%	0%	100%	0%	0%	0%	0%	0%	100%	0%	0%	0%	0%	100%	0%	100%	0%	100%	0%	0%	0%
O6	2020-06-16	hCoV-19/Germany/NRW-MPP-31/2020	protoype	100%	100%	0%	100%	0%	0%	100%	0%	0%	30%	0%	0%	100%	0%	0%	0%	0%	100%	0%	100%	0%	100%	0%	0%	0%
O7	2020-06-17	hCoV-19/Germany/NRW-MPP-32/2020	protoype	100%	100%	0%	100%	0%	0%	100%	0%	0%	0%	0%	0%	100%	28%	0%	0%	0%	100%	0%	100%	0%	100%	0%	0%	0%
O8	2020-06-17	hCoV-19/Germany/NRW-MPP-33/2020	protoype	100%	100%	0%	100%	0%	0%	100%	0%	0%	0%	0%	0%	100%	0%	0%	0%	0%	100%	0%	100%	0%	100%	0%	0%	0%
O9	2020-06-17	hCoV-19/Germany/NRW-MPP-34/2020	variant type 11	100%	100%	100%	100%	0%	62%	100%	0%	0%	0%	0%	0%	99%	0%	0%	0%	0%	100%	0%	100%	0%	100%	0%	0%	0%
O10	2020-06-17	hCoV-19/Germany/NRW-MPP-35/2020	protoype	100%	100%	0%	100%	0%	0%	100%	0%	0%	0%	0%	0%	100%	0%	0%	0%	0%	100%	0%	100%	0%	100%	0%	0%	0%
P1	2020-06-05	hCoV-19/Germany/NRW-MPP-21/2020	protoype	100%	100%	0%	100%	0%	0%	100%	0%	0%	0%	0%	0%	100%	0%	0%	0%	0%	100%	0%	100%	0%	100%	0%	0%	0%
P2	2020-06-05	hCoV-19/Germany/NRW-MPP-22/2020	variant type 6	100%	100%	100%	100%	0%	0%	n.a.	0%	0%	0%	0%	0%	100%	0%	0%	0%	0%	100%	0%	100%	0%	100%	0%	0%	0%
P3	2020-06-08	hCoV-19/Germany/NRW-MPP-23/2020	protoype	100%	100%	0%	100%	0%	0%	100%	0%	0%	0%	0%	0%	100%	0%	0%	0%	0%	100%	0%	100%	0%	100%	0%	0%	0%
P4	2020-06-08	hCoV-19/Germany/NRW-MPP-24/2020	protoype	100%	100%	0%	100%	0%	0%	100%	0%	0%	0%	0%	0%	100%	0%	0%	0%	0%	100%	0%	100%	0%	100%	0%	0%	0%
P5	2020-06-05																											
P6	2020-06-05																											
P7	2020-06-08	hCoV-19/Germany/NRW-MPP-25/2020	variant type 7	100%	100%	0%	100%	0%	0%	100%	0%	0%	0%	0%	0%	100%	0%	100%	0%	0%	100%	0%	100%	0%	100%	0%	0%	0%
D1	2020-05-15	n.d.	protoype	100%	100%	0%	100%	0%	0%	100%	0%	22%	0%	0%	0%	100%	0%	0%	0%	0%	100%	0%	100%	0%	100%	0%	0%	0%
D2	2020-05-15	n.d.	protoype	100%	100%	0%	100%	0%	0%	100%	0%	0%	0%	0%	0%	100%	0%	0%	0%	0%	100%	0%	100%	0%	100%	0%	0%	0%

a Entry ID under which the viral consensus sequence has been submitted to GISAID. Consensus sequences were generated by majority voting at each nucleotide position. Sequences from MPP-D workers (D1, D2) were not submitted to GISAID (n.d.). Blank fields indicate samples for which viral genotyping was not performed.
b Consensus sequence genotype for each entry. The genotype of the index case is designated the prototypic sequence. Genotypes with additional consenus sequence nucleotides are listed as consecutively numbered variant types. Blank fields indicate samples for which viral genotyping was not performed.
c Frequencies of nucleotide variants across samples. n.a. denotes positions at which coverage was insufficient to determine nucleotide sequence. Coding regions and amino acid changes associated with each nucleotide variant are indicated above the table (n.c.: non-coding, n.a.: not available). Blank fields indicate samples for which viral genotyping was not performed.

Appendix Table S2. Infection rates among early shift beef processing workers in relation to workplace distance from the index case.

distance	count[a]	positive count[b]	infection rate	p-value[c]
1	0	0	n.a.	n.a.
2	1	1	100.0%	0.256410
3	4	3	75.0%	0.054464
4	9	5	55.6%	0.054091
5	15	9	60.0%	0.005040
6	19	11	57.9%	0.002848
7	22	14	63.6%	0.000193
8	26	17	65.4%	0.000023
9	29	17	58.6%	0.000171
10	37	18	48.6%	0.002173
11	44	18	40.9%	0.019181
12	48	18	37.5%	0.046932
13	54	18	33.3%	0.128565
14	58	18	31.0%	0.212003
15	62	18	29.0%	0.314127
16	65	18	27.7%	0.398091
17	68	19	27.9%	0.376061
18	70	19	27.1%	0.431501
19	71	19	26.8%	0.459332
20	72	19	26.4%	0.487055
21	72	19	26.4%	0.487055
22	72	19	26.4%	0.487055
23	72	19	26.4%	0.487055
24	72	19	26.4%	0.487055
25	75	19	25.3%	0.568323
26	76	20	26.3%	0.490084
27	76	20	26.3%	0.490084
28	76	20	26.3%	0.490084
29	78	20	25.6%	0.543238

a,b *cumulative counts (excluding the index case) of workes with fixed work stations the indicated distance from the index case*

c *p-value for the hypothesis that the infection rate within the given range is higher than expected by chance*

Appendix Table S3. Infection rates across apartments, bedrooms and carpools shared by early shift beef processing workers.

		unit members				unit members in 8 m area	
unit id	unit type	total count[a]	positive count[b]	infection rate	p-value[c]	count[d]	percentage[e]
a1	apartment	7	5	71.4%	0.037	5	71.4%
a2	apartment	4	3	75.0%	0.098	4	100.0%
a3	apartment	4	2	50.0%	0.379	2	50.0%
a4	apartment	6	1	16.7%	0.899	1	16.7%
a5	apartment	6	3	50.0%	0.289	2	33.3%
a6	apartment	5	1	20.0%	0.852	1	20.0%
a7	apartment	6	1	16.7%	0.899	1	16.7%
a8	apartment	4	0	0.0%	1.000	2	50.0%
a9	apartment	4	3	75.0%	0.098	3	75.0%
a10	apartment	4	0	0.0%	1.000	1	25.0%
a11	apartment	6	0	0.0%	1.000	1	16.7%
r1	bedroom	2	0	0.0%	1.000	0	0.0%
r2	bedroom	2	0	0.0%	1.000	0	0.0%
r3	bedroom	2	0	0.0%	1.000	0	0.0%
r4	bedroom	2	1	50.0%	0.534	1	50.0%
r5	bedroom	3	3	100.0%	0.032	2	66.7%
r6	bedroom	2	1	50.0%	0.534	2	100.0%
r7	bedroom	4	1	25.0%	0.783	0	0.0%
r8	bedroom	2	0	0.0%	1.000	1	50.0%
r9	bedroom	2	1	50.0%	0.534	1	50.0%
r10	bedroom	2	0	0.0%	1.000	1	50.0%
r11	bedroom	2	0	0.0%	1.000	1	50.0%
r12	bedroom	2	2	100.0%	0.101	2	100.0%
r13	bedroom	2	0	0.0%	1.000	1	50.0%
r14	bedroom	2	0	0.0%	1.000	0	0.0%
r15	bedroom	2	0	0.0%	1.000	0	0.0%
r16	bedroom	2	0	0.0%	1.000	1	50.0%
c1	carpool	7	5	71.4%	0.037	5	71.4%
c2	carpool	4	0	0.0%	1.000	1	25.0%
c3	carpool	8	5	62.5%	0.073	6	75.0%
c4	carpool	7	3	42.9%	0.393	6	85.7%
c5	carpool	5	1	20.0%	0.852	1	20.0%
c6	carpool	4	3	75.0%	0.098	3	75.0%
c7	carpool	2	0	0.0%	1.000	1	50.0%
c8	carpool	3	2	66.7%	0.238	1	33.3%
c9	carpool	3	1	33.3%	0.682	2	66.7%

a *count of all members in a given unit*

b *count of infected members in a given unit*

c *p-value for the hypothesis that the infection rate within the given unit is higher than expected by chance*

d,e *count (b) and percentage (c) of unit members with fixed work stations within a maximum distance of 8 meters from the index case*

Appendix Table S4. Primers used in multiplex PCR for Amplicon Sequencing.

Pool 1 Primer ID	sequence	Pool 2 Primer ID	sequence
nCoV-2019_1_LEFT	ACCAACCAACTTTCGATCTCTTGT	nCoV-2019_2_LEFT	CTGTTTTACAGGTTCGCGACGT
nCoV-2019_1_RIGHT	CATCTTTAAGATGTTGACGTGCCTC	nCoV-2019_2_RIGHT	TAAGGATCAGTGCCAAGCTCGT
nCoV-2019_3_LEFT	CGGTAATAAAGGAGCTGGTGGC	nCoV-2019_4_LEFT	GGTGTATACTGCTGCCGTGAAC
nCoV-2019_3_RIGHT	AAGGTGTCTGCAATTCATAGCTCT	nCoV-2019_4_RIGHT	CACAAGTAGTGGCACCTTCTTTAGT
nCoV-2019_5_LEFT	TGGTGAAACTTCATGGCAGACG	nCoV-2019_6_LEFT	GGTGTTGTTGGAGAAGGTTCCG
nCoV-2019_5_RIGHT	ATTGATGTTGACTTTCTCTTTTTGGAGT	nCoV-2019_6_RIGHT	TAGCGGCCTTCTGTAAAACACG
nCoV-2019_7_LEFT_alt0	CATTTGCATCAGAGGCTGCTCG	nCoV-2019_8_LEFT	AGAGTTTCTTAGAGACGGTTGGGA
nCoV-2019_7_RIGHT_alt5	AGGTGACAATTTGTCCACCGAC	nCoV-2019_8_RIGHT	GCTTCAACAGCTTCACTAGTAGGT
nCoV-2019_9_LEFT_alt4	TTCCCACAGAAGTGTTAACAGAGG	nCoV-2019_10_LEFT	TGAGAAGTGCTCTGCCTATACAGT
nCoV-2019_9_RIGHT_alt2	GACAGCATCTGCCACAACACAG	nCoV-2019_10_RIGHT	TCATCTAACCAATCTTCTTCTTGCTCT
nCoV-2019_11_LEFT	GGAATTTGGTGCCACTTCTGCT	nCoV-2019_12_LEFT	AAACATGGAGGAGGTGTTGCAG
nCoV-2019_11_RIGHT	TCATCAGATTCAACTTGCATGGCA	nCoV-2019_12_RIGHT	TTCACTCTTCATTTCCAAAAAGCTTGA
nCoV-2019_13_LEFT	TCGCACAAATGTCTACTTAGCTGT	nCoV-2019_14_LEFT_alt4	TGGCAATCTTCATCCAGATTCTGC
nCoV-2019_13_RIGHT	ACCACAGCAGTTAAAACACCCT	nCoV-2019_14_RIGHT_alt2	TGCGTGTTTCTTCTGCATGTGC
nCoV-2019_15_LEFT_alt1	AGTGCTTAAAAAGTGTAAAAGTGCCT	nCoV-2019_16_LEFT	AATTTGGAAGAAGCTGCTCGGT
nCoV-2019_15_RIGHT_alt3	ACTGTAGCTGGCACTTTGAGAGA	nCoV-2019_16_RIGHT	CACAACTTGCGTGTGGAGGTTA
nCoV-2019_17_LEFT	CTTCTTTCTTTGAGAGAAGTGAGGACT	nCoV-2019_18_LEFT_alt2	ACTTCTATTAAATGGGCAGATAACAACTGT
nCoV-2019_17_RIGHT	TTTGTTGGAGTGTTAACAATGCAGT	nCoV-2019_18_RIGHT_alt1	GCTTGTTTACCACACGTACAAGG
nCoV-2019_19_LEFT	GCTGTTATGTACATGGGCACACT	nCoV-2019_20_LEFT	ACAAAGAAAACAGTTACACAACAACCA
nCoV-2019_19_RIGHT	TGTCCAACTTAGGGTCAATTTCTGT	nCoV-2019_20_RIGHT	ACGTGGCTTTATTAGTTGCATTGTT
nCoV-2019_21_LEFT_alt2	GGCTATTGATTATAAACACTACACACCCT	nCoV-2019_22_LEFT	ACTACCGAAGTTGTAGGAGACATTATACT
nCoV-2019_21_RIGHT_alt0	GATCTGTGTGGCCAACCTCTTC	nCoV-2019_22_RIGHT	ACAGTATTCTTTGCTATAGTAGTCGGC
nCoV-2019_23_LEFT	ACAACTACTAACATAGTTACACGGTGT	nCoV-2019_24_LEFT	AGGCATGCCTTCTTACTGTACTG
nCoV-2019_23_RIGHT	ACCAGTACAGTAGGTTGCAATAGTG	nCoV-2019_24_RIGHT	ACATTCTAACCATAGCTGAAATCGGG
nCoV-2019_25_LEFT	GCAATTGTTTTTCAGCTATTTTGCAGT	nCoV-2019_26_LEFT	TTGTGATACATTCTGTGCTGGTAGT
nCoV-2019_25_RIGHT	ACTGTAGTGACAAGTCTCTCGCA	nCoV-2019_26_RIGHT	TCCGCACTATCACCAACATCAG
nCoV-2019_27_LEFT	ACTACAGTCAGCTTATGTGTCAACC	nCoV-2019_28_LEFT	ACATAGAAGTTACTGGCGATAGTTGT
nCoV-2019_27_RIGHT	AATACAAGCACCAAGGTCACGG	nCoV-2019_28_RIGHT	TGTTTAGACATGACATGAACAGGTGT
nCoV-2019_29_LEFT	ACTTGTGTTCCTTTTTGTTGCTGC	nCoV-2019_30_LEFT	GCACAACTAATGGTGACTTTTTGCA
nCoV-2019_29_RIGHT	AGTGTACTCTATAAGTTTTGATGGTGTGT	nCoV-2019_30_RIGHT	ACCACTAGTAGATACACAAACACCAG
nCoV-2019_31_LEFT	TTCTGAGTACTGTAGGCACGGC	nCoV-2019_32_LEFT	TGGTGAATACAGTCATGTAGTTGCC
nCoV-2019_31_RIGHT	ACAGAATAAACACCAGGTAAGAATGAGT	nCoV-2019_32_RIGHT	AGCACATCACTACGCAACTTTAGA
nCoV-2019_33_LEFT	ACTTTTGAAGAAGCTGCGCTGT	nCoV-2019_34_LEFT	TCCCATCTGGTAAAGTTGAGGGT
nCoV-2019_33_RIGHT	TGGACAGTAAACTACGTCATCAAGC	nCoV-2019_34_RIGHT	AGTGAAATTGGGCCTCATAGCA
nCoV-2019_35_LEFT	TGTTCGCATTCAACCAGGACAG	nCoV-2019_36_LEFT	TTAGCTTGGTTGTACGCTGCTG
nCoV-2019_35_RIGHT	ACTTCATAGCCACAAGGTTAAAGTCA	nCoV-2019_36_RIGHT	GAACAAAGACCATTGAGTACTCTGGA
nCoV-2019_37_LEFT	ACACACCACTGGTTGTTACTCAC	nCoV-2019_38_LEFT	ACTGTGTTATGTATGCATCAGCTGT
nCoV-2019_37_RIGHT	GTCCACACTCTCCTAGCACCAT	nCoV-2019_38_RIGHT	CACCAAGAGTCAGTCTAAAGTAGCG
nCoV-2019_39_LEFT	AGTATTGCCCTATTTTCTTCATAACTGGT	nCoV-2019_40_LEFT	TGCACATCAGTAGTCTTACTCTCAGT
nCoV-2019_39_RIGHT	TGTAACTGGACACATTGAGCCC	nCoV-2019_40_RIGHT	CATGGCTGCATCACGGTCAAAT
nCoV-2019_41_LEFT	GTTCCCTTCCATCATATGCAGCT	nCoV-2019_42_LEFT	TGCAAGAGATGGTTGTGTTCCC
nCoV-2019_41_RIGHT	TGGTATGACAACCATTAGTTTGGCT	nCoV-2019_42_RIGHT	CCTACCTCCCTTTGTTGTGTTGT
nCoV-2019_43_LEFT	TACGACAGATGTCTTGTGCTGC	nCoV-2019_44_LEFT_alt3	CCACAGTACGTCTACAAGCTGG
nCoV-2019_43_RIGHT	AGCAGCATCTACAGCAAAAGCA	nCoV-2019_44_RIGHT_alt0	CGCAGACGGTACAGACTGTGTT
nCoV-2019_45_LEFT_alt2	AGTATGTACAAATACCTACAACTTGTGCT	nCoV-2019_46_LEFT_alt1	CGCTTCCAAGAAAAGGACGAAGA
nCoV-2019_45_RIGHT_alt7	TTCATGTTGGTAGTTAGAGAAAGTGTGTC	nCoV-2019_46_RIGHT_alt2	CACGTTCACCTAAGTTGGCGTAT
nCoV-2019_47_LEFT	AGGACTGGTATGATTTTGTAGAAAACCC	nCoV-2019_48_LEFT	TGTTGACACTGACTTAACAAAGCCT
nCoV-2019_47_RIGHT	AATAACGGTCAAAGAGTTTTAACCTCTC	nCoV-2019_48_RIGHT	TAGATTACCAGAAGCAGCGTGC
nCoV-2019_49_LEFT	AGGAATTACTTGTGTATGCTGCTGA	nCoV-2019_50_LEFT	GTTGATAAGTACTTTGATTGTTACGATGGT
nCoV-2019_49_RIGHT	TGACGATGACTTGGTTAGCATTAATACA	nCoV-2019_50_RIGHT	TAACATGTTGTGCCAACCACCA
nCoV-2019_51_LEFT	TCAATAGCCGCCACTAGAGGAG	nCoV-2019_52_LEFT	CATCAGGAGATGCCACAACTGC
nCoV-2019_51_RIGHT	AGTGCATTAACATTGGCCGTGA	nCoV-2019_52_RIGHT	GTTGAGAGCAAAATTCATGAGGTCC
nCoV-2019_53_LEFT	AGCAAAATGTTGGACTGAGACTGA	nCoV-2019_54_LEFT	TGAGTTAACAGGACACATGTTAGACA
nCoV-2019_53_RIGHT	AGCCTCATAAAACTCAGGTTCCC	nCoV-2019_54_RIGHT	AACCAAAAACTTGTCCATTAGCACA
nCoV-2019_55_LEFT	ACTCAACTTTACTTAGGAGGTATGAGCT	nCoV-2019_56_LEFT	ACCTAGACCACCACTTAACCGA
nCoV-2019_55_RIGHT	GGTGTACTCTCCTATTTGTACTTTACTGT	nCoV-2019_56_RIGHT	ACACTATGCGAGCAGAAGGGTA
nCoV-2019_57_LEFT	ATTCTACACTCCAGGGACCACC	nCoV-2019_58_LEFT	TGATTTGAGTGTTGTCAATGCCAGA
nCoV-2019_57_RIGHT	GTAATTGAGCAGGGTCGCCAAT	nCoV-2019_58_RIGHT	CTTTTCTCCAAGCAGGGTTACGT
nCoV-2019_59_LEFT	TCACGCATGATGTTTCATCTGCA	nCoV-2019_60_LEFT	TGATAGAGACCTTTATGACAAGTTGCA
nCoV-2019_59_RIGHT	AAGAGTCCTGTTACATTTTCAGCTTG	nCoV-2019_60_RIGHT	GGTACCAACAGCTTCTCTAGTAGC
nCoV-2019_61_LEFT	TGTTTATCACCCGCGAAGAAGC	nCoV-2019_62_LEFT	GGCACATGGCTTTGAGTTGACA
nCoV-2019_61_RIGHT	ATCACATAGACAACAGGTGCGC	nCoV-2019_62_RIGHT	GTTGAACCTTTCTACAAGCCGC
nCoV-2019_63_LEFT	TGTTAAGCGTGTTGACTGGACT	nCoV-2019_64_LEFT	TCGATAGATATCCTGCTAATTCCATTGT
nCoV-2019_63_RIGHT	ACAAACTGCCACCATCACAACC	nCoV-2019_64_RIGHT	AGTCTTGTAAAAGTGTTCCAGAGGT
nCoV-2019_65_LEFT	GCTGGCTTTAGCTTGTGGGTTT	nCoV-2019_66_LEFT	GGGTGTGGACATTGCTGCTAAT
nCoV-2019_65_RIGHT	TGTCAGTCATAGAACAAACACCAATAGT	nCoV-2019_66_RIGHT	TCAATTTCCATTTGACTCCTGGGT
nCoV-2019_67_LEFT	GTTGTCCAACAATTACCTGAAACTTACT	nCoV-2019_68_LEFT	ACAGGTTCATCTAAGTGTGTGTGT
nCoV-2019_67_RIGHT	CAACCTTAGAAACTACAGATAAATCTTGGG	nCoV-2019_68_RIGHT	CTCCTTTATCAGAACCAGCACCA
nCoV-2019_69_LEFT	TGTCGCAAAATATACTCAACTGTGTCA	nCoV-2019_70_LEFT	ACAAAAGAAAATGACTCTAAAGAGGGTTT
nCoV-2019_69_RIGHT	TCTTTATAGCCACGGAACCTCCA	nCoV-2019_70_RIGHT	TGACCTTCTTTTAAAGACATAACAGCAG
nCoV-2019_71_LEFT	ACAAATCCAATTCAGTTGTCTTCCTATTC	nCoV-2019_72_LEFT	ACACGTGGTGTTTATTACCCTGAC

nCoV-2019_71_RIGHT	TGGAAAAGAAAGGTAAGAACAAGTCCT	nCoV-2019_72_RIGHT	ACTCTGAACTCACTTTCCATCCAAC
nCoV-2019_73_LEFT	CAATTTTGTAATGATCCATTTTTGGGTGT	nCoV-2019_74_LEFT	ACATCACTAGGTTTCAAACTTTACTTGC
nCoV-2019_73_RIGHT	CACCAGCTGTCCAACCTGAAGA	nCoV-2019_74_RIGHT	GCAACACAGTTGCTGATTCTCTTC
nCoV-2019_75_LEFT	AGAGTCCAACCAACAGAATCTATTGT	nCoV-2019_76_LEFT_alt3	GGGCAAACTGGAAAGATTGCTGA
nCoV-2019_75_RIGHT	ACCACCAACCTTAGAATCAAGATTGT	nCoV-2019_76_RIGHT_alt0	ACCTGTGCCTGTTAAACCATTGA
nCoV-2019_77_LEFT	CCAGCAACTGTTTGTGGACCTA	nCoV-2019_78_LEFT	CAACTTACTCCTACTTGGCGTGT
nCoV-2019_77_RIGHT	CAGCCCCTATTAAACAGCCTGC	nCoV-2019_78_RIGHT	TGTGTACAAAAACTGCCATATTGCA
nCoV-2019_79_LEFT	GTGGTGATTCAACTGAATGCAGC	nCoV-2019_80_LEFT	TTGCCTTGGTGATATTGCTGCT
nCoV-2019_79_RIGHT	CATTTCATCTGTGAGCAAAGGTGG	nCoV-2019_80_RIGHT	TGGAGCTAAGTTGTTTAACAAGCG
nCoV-2019_81_LEFT	GCACTTGGAAAACTTCAAGATGTGG	nCoV-2019_82_LEFT	GGGCTATCATCTTATGTCCTTCCCT
nCoV-2019_81_RIGHT	GTGAAGTTCTTTTCTTGTGCAGGG	nCoV-2019_82_RIGHT	TGCCAGAGATGTCACCTAAATCAA
nCoV-2019_83_LEFT	TCCTTTGCAACCTGAATTAGACTCA	nCoV-2019_84_LEFT	TGCTGTAGTTGTCTCAAGGGCT
nCoV-2019_83_RIGHT	TTTGACTCCTTTGAGCACTGGC	nCoV-2019_84_RIGHT	AGGTGTGAGTAAACTGTTACAAACAAC
nCoV-2019_85_LEFT	ACTAGCACTCTCCAAGGGTGTT	nCoV-2019_86_LEFT	TCAGGTGATGGCACAACAAGTC
nCoV-2019_85_RIGHT	ACACAGTCTTTTACTCCAGATTCCC	nCoV-2019_86_RIGHT	ACGAAAGCAAGAAAAAGAAGTACGC
nCoV-2019_87_LEFT	CGACTACTAGCGTGCCTTTGTA	nCoV-2019_88_LEFT	CCATGGCAGATTCCAACGGTAC
nCoV-2019_87_RIGHT	ACTAGGTTCCATTGTTCAAGGAGC	nCoV-2019_88_RIGHT	TGGTCAGAATAGTGCCATGGAGT
nCoV-2019_89_LEFT_alt2	CGCGTTCCATGTGGTCATTCAA	nCoV-2019_90_LEFT	ACACAGACCATTCCAGTAGCAGT
nCoV-2019_89_RIGHT_alt4	ACGAGATGAAACATCTGTTGTCACT	nCoV-2019_90_RIGHT	TGAAATGGTGAATTGCCCTCGT
nCoV-2019_91_LEFT	TCACTACCAAGAGTGTGTTAGAGGT	nCoV-2019_92_LEFT	TTTGTGCTTTTTAGCCTTTCTGCT
nCoV-2019_91_RIGHT	TTCAAGTGAGAACCAAAAGATAATAAGCA	nCoV-2019_92_RIGHT	AGGTTCCTGGCAATTAATTGTAAAAGG
nCoV-2019_93_LEFT	TGAGGCTGGTTCTAAATCACCCA	nCoV-2019_94_LEFT	GGCCCCAAGGTTTACCCAATAA
nCoV-2019_93_RIGHT	AGGTCTTCCTTGCCATGTTGAG	nCoV-2019_94_RIGHT	TTTGGCAATGTTGTTCCTTGAGG
nCoV-2019_95_LEFT	TGAGGGAGCCTTGAATACACCA	nCoV-2019_96_LEFT	GCCAACAACAACAAGGCCAAAC
nCoV-2019_95_RIGHT	CAGTACGTTTTTGCCGAGGCTT	nCoV-2019_96_RIGHT	TAGGCTCTGTTGGTGGGAATGT
nCoV-2019_97_LEFT	TGGATGACAAAGATCCAAATTTCAAAGA	nCoV-2019_98_LEFT	AACAATTGCAACAATCCATGAGCA
nCoV-2019_97_RIGHT	ACACACTGATTAAAGATTGCTATGTGAG	nCoV-2019_98_RIGHT	TTCTCCTAAGAAGCTATTAAAATCACATGG

Appendix Table S5. Amplicon-Seq statistics.

sample ID	rep#*	read counts	coverage (average)**	stdev	coverage (median) **	25th percentile	50th percentile	75th percentile	covered (%)**
B1	1	31466	362.7	196.3	345	228	345	458	100.0
B1	2	29502	340.0	178.3	314	217	314	433	100.0
B2	1	24165	278.7	163.2	286	162	286	353	100.0
B2	2	32248	371.6	220.9	362	199	362	502	100.0
B5	1	27837	321.0	219.5	301	143	301	440	100.0
B5	2	33036	380.6	237.1	365	208	365	524	100.0
B6	1	27594	318.0	191.9	306	179	306	418	100.0
B6	2	26723	307.7	182.7	284	179	284	416	100.0
B7	1	28782	331.6	218.3	322	166	322	463	100.0
B7	2	26853	309.6	206.7	299	150	299	424	100.0
B14	1	33212	384.8	549.1	159	24	159	463	92.0
B24	1	33485	386.1	223.2	378	240	378	492	100.0
B24	2	23302	268.5	150.5	256	157	256	341	100.0
B55	1	28897	332.6	172.5	321	221	321	404	100.0
B55	2	23373	269.1	140.8	251	181	251	335	100.0
B57	1	31869	367.0	224.7	340	218	340	484	100.0
B57	2	23027	265.3	159.4	242	152	242	336	100.0
B72	1	26706	307.9	208.2	315	140	315	423	100.0
B72	2	25333	292.0	194.5	292	129.75	292	397	100.0
B76	1	21963	253.2	154.8	228	154	228	339	100.0
B76	2	26611	306.4	170.7	281	187	281	390	100.0
B80	1	23140	266.7	214.8	245	86	245	390	100.0
B80	2	28317	326.5	228.7	313	138	313	469	100.0
B81	1	23412	270.2	220.2	260	94	260	390	98.2
B81	2	26996	311.1	214.5	311	144	311	439	100.0
B83	1	28699	331.1	287.8	297	100	297	498	99.0
B83	2	29652	342.0	262.8	300	135	300	490	99.2
B90	1	26181	302.9	320.6	223	40	223	433	97.4
B90	2	27205	314.5	293.2	256	92	256	470	97.4
B93	1	24823	286.2	169.8	274	163	274	386	100.0
B93	2	22981	264.6	158.1	241	148	241	352	100.0
B98	1	31366	362.1	283.3	364	144	364	498	100.0
B98	2	25067	289.1	193.6	295	150	295	397	100.0
B114	1	26101	300.6	255.6	256	90	256	459	100.0
B114	2	29577	341.1	219.5	351	184	351	472	100.0
B121	1	33882	391.2	276.3	406	204	406	551	100.0
B121	2	32130	370.7	253.5	380	182	379.5	501	100.0
B148	1	26283	302.7	200.5	289	162	289	414	100.0
B148	2	24778	285.2	171.0	271	165	270.5	384	100.0
O1	1	68603	793.9	944.9	368	52	368	1148	98.2
O1	2	58107	673.0	849.4	293	33	293	912	95.7
O2	1	77839	897.7	528.9	895	479	895	1175	100.0
O2	2	60383	696.4	398.8	684	407	684	889	100.0
O3	1	69961	807.0	401.3	765	529	765	1027	100.0
O3	2	58416	673.8	344.4	646	429	646	846	100.0
O4	1	84575	976.8	713.5	952	354	952	1443	100.0
O4	2	82025	947.0	708.3	863	303	863	1386	100.0
O5	1	56255	648.8	308.0	624	450	624	810	100.0
O5	2	27286	313.9	163.8	285	205	285	393	100.0
O6	1	74388	860.2	842.2	662	145	662	1258	99.2
O6	2	74010	854.9	764.8	675	169	675	1345	99.2
O7	1	54104	623.6	289.2	582	410	582	768	100.0
O7	2	77352	891.1	413.8	806	637	806	1074	100.0
O8	1	69675	804.5	541.0	814	360	814	1110	100.0
O8	2	67667	780.0	435.0	795	489	795	1022	100.0

O9	1	56886	656.1	351.7	650	414	650	853	100.0
O9	2	74283	856.8	467.4	864	515	864	1071	100.0
O10	1	62298	719.4	571.4	642	229	642	1006	99.2
O10	2	50281	580.9	433.2	540	163	540	875	100.0
P1	1	74427	859.5	645.4	790	301	790	1243	100.0
P1	2	70738	816.5	564.5	805	372	805	1190	100.0
P2	1	74485	862.7	1436.8	164	20	164	805	94.7
P2	2	89809	1041.5	1671.6	217	24	217	1153	96.4
P3	1	58514	674.7	361.3	651	416	651	858	100.0
P3	2	53533	617.4	322.0	603	390	603	797	100.0
P4	1	55782	644.5	412.5	642	292	642	920	100.0
P4	2	81385	939.1	613.2	949	445	949	1285	100.0
P7	1	63529	732.7	368.4	714	493	714	912	100.0
P7	2	71523	825.9	371.8	812	621	812	978	100.0
D1	1	132841	1535.6	940.9	1490	846	1490	2113	100.0
D1	2	117432	1354.9	605.6	1299	984	1299	1646	100.0
D2	1	117149	1352.2	822.5	1269	800	1269	1812	100.0
D2	2	161684	1866.5	820.4	1740	1399	1740	2177	100.0

*) Replicates represent independent cDNA synthesis, library preparation and sequencing reactions performed from the same RNA sample.

**) calculated for the part of the genome that is targeted by amplicon-seq (nt. 55-29836).

Appendix Table 6. Comparison of SARS-CoV-2 genotypes identified in this study to sequences deposited in the GISAID database

strain	gisaid accession	date	country	division	clade	C6406T	C18972A	date submitted
England/CAMB-782FE/2020	EPI_ISL_433715	2020-04-04	UK	England	20B	no	yes	2020-04-29
Wales/PHWC-15D09A/2020	EPI_ISL_472664	2020-05-03	UK	Wales	20B	yes	no	2020-06-23
Wales/PHWC-15D188/2020	EPI_ISL_472676	2020-04-19	UK	Wales	20B	yes	no	2020-06-23
Wales/PHWC-15DC78/2020	EPI_ISL_472768	2020-05-13	UK	Wales	20B	yes	no	2020-06-23
Wales/PHWC-16093F/2020	EPI_ISL_472995	2020-05-22	UK	Wales	20B	yes	no	2020-06-23
Wales/PHWC-16344A/2020	EPI_ISL_473296	2020-05-06	UK	Wales	20B	yes	no	2020-06-23
Wales/PHWC-163592/2020	EPI_ISL_473965	2020-04-24	UK	Wales	20B	yes	no	2020-06-23
Wales/PHWC-163BEB/2020	EPI_ISL_474055	2020-05-09	UK	Wales	20B	yes	no	2020-06-23
Wales/PHWC-163E4C/2020	EPI_ISL_474091	2020-05-06	UK	Wales	20B	yes	no	2020-06-23
Wales/PHWC-163F67/2020	EPI_ISL_474107	2020-04-22	UK	Wales	20B	yes	no	2020-06-23
Wales/PHWC-164F1B/2020	EPI_ISL_479398	2020-05-18	UK	Wales	20B	yes	no	2020-06-30
Wales/PHWC-2ACA2/2020	EPI_ISL_445688	2020-04-06	UK	Wales	20B	yes	no	2020-05-16
Wales/PHWC-2E312/2020	EPI_ISL_446088	2020-04-16	UK	Wales	20B	yes	no	2020-05-16
Wales/PHWC-31B6F/2020	EPI_ISL_474326	2020-04-11	UK	Wales	20B	no	yes	2020-06-23
Wales/PHWC-31BE7/2020	EPI_ISL_446542	2020-04-11	UK	Wales	20B	yes	no	2020-05-16
Wales/PHWC-323C0/2020	EPI_ISL_446641	2020-04-08	UK	Wales	20B	yes	no	2020-05-16
Wales/PHWC-33101/2020	EPI_ISL_446739	2020-04-14	UK	Wales	20B	yes	no	2020-05-16
Wales/PHWC-34267/2020	EPI_ISL_446892	2020-04-18	UK	Wales	20B	yes	no	2020-05-16
Wales/PHWC-34926/2020	EPI_ISL_446995	2020-04-18	UK	Wales	20B	yes	no	2020-05-16
Wales/PHWC-35A7D/2020	EPI_ISL_474519	2020-04-25	UK	Wales	20B	yes	no	2020-06-23
Wales/PHWC-369E8/2020	EPI_ISL_474744	2020-04-24	UK	Wales	20B	yes	no	2020-06-23
USA/CA-CZB-1320/2020	EPI_ISL_468354	2020-05-20	USA	California	20C	yes	no	2020-06-16
USA/CA-CZB-1322/2020	EPI_ISL_468355	2020-05-20	USA	California	20C	yes	no	2020-06-16
